# Comparison of Conventional Imaging and 18F-Fluorodeoxyglucose Positron Emission Tomography/Computed Tomography in the Diagnostic Accuracy of Staging in Patients with Intrahepatic Cholangiocarcinoma

**DOI:** 10.3390/diagnostics12112889

**Published:** 2022-11-21

**Authors:** Eiko Nishioka, Masakatsu Tsurusaki, Ryohei Kozuki, Sung-Woon Im, Atsushi Kono, Kazuhiro Kitajima, Takamichi Murakami, Kazunari Ishii

**Affiliations:** 1 Department of Radiology, Kobe University Graduate School of Medicine, Kobe 650-0017, Hyogo, Japan; 2 Department of Radiology, Kindai University Faculty of Medicine, Osaka-Sayama 589-8511, Osaka, Japan; 3 Department of Radiology, Hyogo Medical University Faculty of Medicine, Nishinomiya 663-8501, Hyogo, Japan

**Keywords:** intrahepatic cholangiocarcinoma, 18F-fluorodeoxyglucose positron emission tomography, computer tomography, magnetic resonance imaging, tumor stage

## Abstract

We aimed to examine the accuracy of tumor staging of intrahepatic cholangiocarcinoma (ICC) by using 18F-fluorodeoxyglucose positron emission tomography/computed tomography (18F-FDG PET-CT). From January 2001 to December 2021, 202 patients underwent PET-CT, CT, and MRI for the initial staging of ICC in two institutions. Among them, 102 patients had undergone surgical treatment. Ninety patients who had a histopathological diagnosis of ICC were retrospectively reviewed. The sensitivity and specificity of 18F-FDG PET-CT, CT, and magnetic resonance imaging (MRI) in detecting tumors, satellite focus, vascular invasion, and lymph node metastases were analyzed. Ninety patients with histologically diagnosed ICC were included. PET-CT demonstrated no statistically significant advantage over CT and MR in the diagnosis of multiple tumors and macrovascular invasion, and bile duct invasion. The overall sensitivity, specificity, positive predictive value (PPV), negative predictive value (NPV), and accuracy of PET-CT in lymph node metastases were 84%, 86%, 91%, 84%, and 86%, respectively. PET-CT revealed a significantly higher accuracy compared to CT or MRI (86%, 67%, and 76%, *p* < 0.01, respectively) in the diagnosis of regional lymph node metastases. The accuracy of tumor staging by PET-CT was higher than that by CT/MRI (PET-CT vs. CT vs. MRI: 68/90 vs. 47/90 vs. 51/90, *p* < 0.05). 18F-FDG PET-CT had sensitivity and specificity values for diagnosing satellite focus and vascular and bile duct invasion similar to those of CT or MRI; however, PET-CT showed higher accuracy in diagnosing regional lymph node metastases. 18F-FDG PET-CT exhibited higher tumor staging accuracy than that of CT/MRI. Thus, 18FDG PET-CT may support tumor staging in ICC.

## 1. Introduction

Intrahepatic cholangiocarcinoma (ICC) is the second most common primary hepatic malignancy after hepatocellular carcinoma (HCC). Epidemiological studies show that ICC is rare in most western countries. However, the incidence is exceptionally high in Asian countries, such as China, Korea, Japan, and Thailand. The northeastern region of Thailand has the highest incidence worldwide (85 per 100,000 population/year) [1]. Notably, ICC shares some common risk factors with HCC, such as hepatitis B or C viral infection, cirrhosis, diabetes, and alcohol abuse [2]. The early diagnosis of ICC is difficult, as the disease is typically asymptomatic. The tumor, nodes, and metastases (TNM) system by the International Union Against Cancer (UICC)/American Joint Committee on Cancer (AJCC) is widely used for ICC staging. However, until the sixth edition of this staging system, the staging of HCC and ICC was identical, with the distinction between these two only being introduced in the seventh edition and updated in the current eighth edition. [3]. Computed tomography/magnetic resonance imaging (CT/MRI) is routinely used in the diagnosis and staging of ICC. However, the associated diagnostic accuracy, sensitivity, specificity, and TNM staging are considered suboptimal for ICC management [4,5].

Positron emission tomography (PET) using 18F-fluorodeoxyglucose (18F-FDG) is an imaging modality that measures the glucose metabolism rate in tumor cells and thus provides biochemical information about tumors, which is not provided by other imaging modalities. Increased uptake of 18F-FDG represents an enhanced glucose metabolism in cancer cells, which makes it a marker of tumor viability. Expectedly, 18F-FDG PET-CT is increasingly used in the diagnosis, prognosis, staging, and treatment monitoring of many tumor types [6,7,8,9,10,11]. To our knowledge, there has been only one meta-analysis, and few studies have evaluated the diagnostic efficacy of 18F-FDG PET/CT for patients with ICC [12,13,14,15,16,17], which is not currently included in the routine clinical management of ICC [1,18,19]. There was no study that evaluated the correlation among associated diagnostic accuracy, sensitivity, specificity, and the eighth edition of TNM staging for ICC.

Our preliminary findings suggest that this modality may help identify lymph nodes and distant metastases in patients with ICC. Therefore, a cohort study of patients with ICC was conducted to compare the efficacy of 18F-FDG PET-CT with that of CT/MRI in tumor staging.

## 2. Materials and Methods

### 2.1. Patients

Patients who were diagnosed with ICC using CT/MRI at the Kindai University Hospital and Hyogo Medical University Hospital between January 2001 and December 2021 were retrospectively reviewed. These patients were routinely investigated with conventional radiological imaging modalities, such as chest radiography/CT and abdominal CT/MRI, to assess the tumor stage. Simultaneously, 18F-FDG PET-CT was performed for these patients. All imaging examinations were performed 4 weeks before treatment. The patient’s demographic and clinical characteristics data were collected, and the TNM stage was assessed using the eighth edition of the AJCC/Union for International Cancer Control (UICC) staging system [3]. The study was conducted in accordance with the Declaration of Helsinki. Approval was granted by the local ethics committee (25-117). The requirement for informed consent was waived.

### 2.2. Conventional Radiological Imaging

Contrast-enhanced abdominal CT (Aquilion Multi 64, Toshiba Medical Systems, Otawara, Japan, and Discovery CT 750HD, GE Medical Systems, Milwaukee, WI, USA) and MRI at a 3T scanner (Intera Acheiva 3T, Philips Healthcare, Best, The Netherlands) or 1.5T scanner (1.5T Signa HDxt, GE Healthcare, Milwaukee, WI, USA) were used to evaluate the primary tumors and to detect any intraabdominal metastases. Chest radiography and whole-body bone scintigraphy were used to identify distant metastases. After the injection of intravenous contrast (150 mL at 3 mL/s), CT and MRI scans of the abdomen were obtained with 5 mm collimation and a table speed of 5 mm/s. The images were reconstructed with 5 mm thickness. The CT and MRI scans were interpreted by radiologists with over 10 years of experience. A diagnosis of ICC was defined as an irregular mass with markedly low attenuation, peripheral rim enhancement followed by the progressive and concentric filling with contrast, and the focal dilatation of the intrahepatic ducts around the tumor. Enlarged lymph nodes were identified on CT/MRI and were measured along their short axes. Lymph node metastasis was defined as a short axis of ≥10 mm or when it increased by ≥20% on sequential CT/MRI scans within an interval of four weeks [20].

### 2.3. PET

The 18F-FDG PET-CT images were obtained using a PET/CT scanner (Discovery PET/CT 710; GE Healthcare Life Sciences, Amersham Place, Little Chalfont, Buckinghamshire, England). The patients fasted for at least four hours before the intravenous administration of 3.0 MBq/kg of 18F-FDG (80.7–307.5 MBq). The blood sugar level was checked before administration, and none of the patients had a glucose level greater than 115 mg/dL. The patients underwent full-body scans. Imaging began approximately 60 min after an intravenous administration of 18F-FDG using the 3D time-of-flight mode (3D TOF), and the helical CT data acquired with free breathing were used for attenuation correction. The CT scanning parameters were 120 kV, an automated tube current with a noise index of 23 for helical CT, 0.5 s/rotation, a pitch factor of 1.375, a detector configuration of 16 × 1.25 mm, a slice thickness of 3.75 mm, a slice interval of 3.27 mm, and a display field-of-view (DFOV) of 500 mm. All PET images were reconstructed using a block sequential regularized expectation maximization algorithm. We adopted a β value of 800. The PET imaging properties were as follows: a slice thickness and interval of 3.27 mm and a matrix size of 192 × 192, and a DFOV of 500 mm. Accurate patient positioning between the transmission and emission scanning was performed via laser marking. Both attenuation-corrected and non-attenuation-corrected images were interpreted visually. The attenuation-corrected images were then analyzed semi-quantitatively using standard uptake values (SUV). Regions of interest were drawn over the area of maximum activity in a lesion. The SUV was calculated as follows: SUV = tissue concentration/(injected dose × body weight). A primary tumor was defined when a focal lesion with an increase in the 18F-FDG uptake was detected relative to the values observed in the surrounding normal tissue. A lymph node was defined as positive when SUVmax was ≥2.5 [20].

### 2.4. Statistical Analysis

Categorical variables were presented as numbers (percentages) and compared using the χ^2^ test. Continuous variables were checked for normality, and continuous variables were presented as the mean ± standard deviation (SD) and compared using the *t*-test. The McNemar test was used to compare the diagnostic performance between PET/CT and conventional imaging methods. These analyses were performed using SPSS Statistics version 17.0 (SPSS; IBM, Tokyo, Japan). *p*-values < 0.05 were considered statistically significant.

## 3. Results

From January 2001 to December 2021, 255 consecutive patients were clinically diagnosed with ICC. Among them, 202 patients underwent PET-CT, CT, and MRI during their preoperative diagnosis session, including 102 patients who had undergone surgical treatment. Among the 102 patients who had a histopathological diagnosis, 90 had ICC and were included in this study (Table 1). The tumor size, tumor number, macroscopic vascular invasion, cancer embolus in the bile duct, lymph node metastases, nerve invasion, and peripheral tissue invasion were evaluated by histopathological examinations (Supplementary Figure S1).

Eleven patients were diagnosed histopathologically with HCC and one with an isolated necrotic nodule. Among 90 patients with ICC, 85 underwent hepatectomy plus lymphadenectomy, and five underwent exploratory laparotomy only because of the intraoperative detection of abdominal metastases (peritoneal dissemination). Moreover, 105 cases (including five exploratory laparotomy cases) were considered unresectable preoperatively due to multiple tumors in the future remnant liver (n = 10), peritoneal dissemination (n = 5), distant lymph node metastasis (n = 48), and distant organ metastases (n = 42). Among the 90 patients that underwent surgery, 35 patients had multiple tumors, 5 had vascular invasion, 6 had bile duct invasion, and 25 had regional lymph node metastases (Table 1).

Multiple tumors were diagnosed by CT, MRI, and PET-CT scans with corresponding accuracy rates of 80%, 79%, and 72%, respectively. The diagnosis of vascular invasion in patients by CT, MRI, and PET-CT had accuracy rates of 88%, 92%, and 94%, respectively. Bile duct invasion was diagnosed by CT, MRI, and PET-CT and had corresponding accuracy rates of 93%, 96%, and 94%, respectively. Twenty-five patients had histologically confirmed metastases in the regional lymph nodes. Regional lymph node metastases detected by CT had a sensitivity of 40%, MRI had a sensitivity of 56%, and PET-CT had a sensitivity of 84%. These results are shown in detail in Table 2.

The corresponding specificity rates were 80%, 83%, and 86%, respectively. PET-CT showed higher accuracy (86%) than those of CT (67%) and MRI (76%) at diagnosing lymph node metastasis in patients with ICC (*p* < 0.01). Among the 34 patients with lymph nodes > 1 cm, 23 (68%) patients had lymph node metastases. Among the 23 patients with lymph nodes of ≤1 cm, 2 (8.7%) were positive for metastases. There was a positive association between the lymph node metastases status and lymph node size (*p* < 0.01).

The TNM staging of each patient was assessed using PET-CT or conventional radiological imaging, including abdominal CT or MRI, chest radiography, and whole-body bone scintigraphy (CT/MRI) evaluations (Table 3). Among them, 33/30/19 patients were down-staged, and 10/9/3 were up-staged by CT/MRI/PET-CT when compared with pathological examination. The accuracy of tumor staging by PET-CT was higher than that by CT/MRI (PET-CT vs. CT vs. MRI: 68/90 vs. 47/90 vs. 51/90, *p* = 0.003) (Table 4). PET-CT had no impact on patient management in 76% of cases.

## 4. Discussion

**Figure 1:**
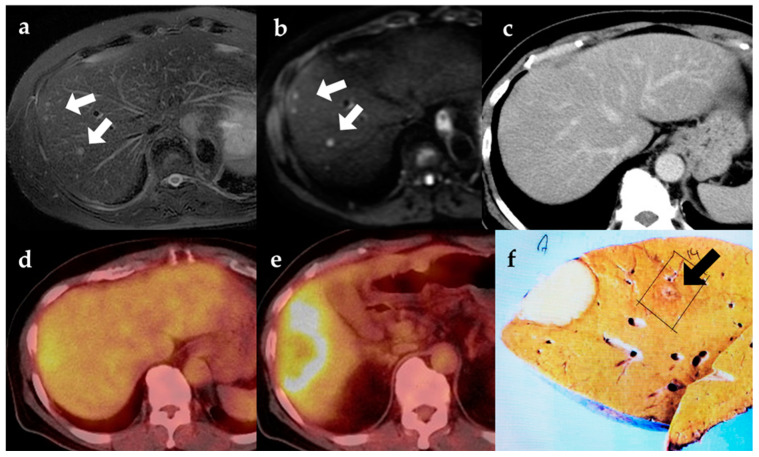
MRI (**a**,**b**) showed a satellite nodule (white arrow) separated from the main tumor, which was confirmed by histopathology ((**f**); black arrow) but was not detected on CT (**c**) or PET-CT (**d**,**e**). (**a**) T2-weighted MR image, (**b**) Diffusion-weighted MR image (b factor = 800), (**c**) Contrast-enhanced CT, (**d**) PET-CT, (**e**) PET-CT showing main tumor, (**f**) Gross specimen.

**Figure 2:**
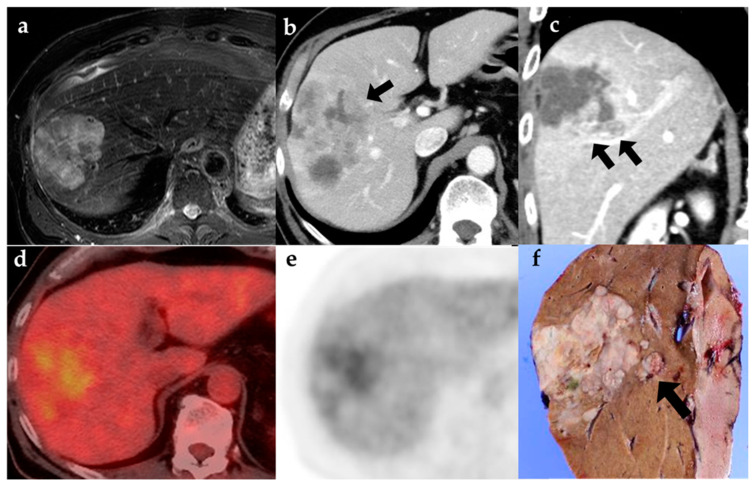
Contrast-enhanced CT showed bile duct ((**b**); arrow) and vascular emboli ((**c**); arrow), which were not detected on MRI (**a**) or PET-CT (**d**,**e**). (**a**) T2-weighted MR image, (**b**) Axial section of contrast-enhanced CT, (**c**) Coronal section of contrast-enhanced CT (**d**,**e**) PET-CT, (**f**) Gross specimen (vascular emboli; arrow).

The 18F-FDG PET scan is a radiological technique that combines anatomic and functional imaging. It is increasingly used in the management of different tumors [21,22,23,24,25,26]. Most studies find it valuable in diagnosing and staging tumors when compared with traditional imaging techniques such as CT and MRI. There were a few studies presenting the advantages and disadvantages of 18F-FDG PET-CT with a particular focus on ICC. It has also been reported as useful in malignant biliary cancer, including ICC, as it outperformed CT and MRI at detecting lymph node metastases [13,14,20]. Two meta-analyses compared the diagnostic value of 18F-FDG PET/CT versus MRI for the staging of both extrahepatic and intrahepatic cholangiocarcinoma (ECC/ICC) [27,28]. This meta-analysis indicated that both MRI and 18F-FDG PET/CT could provide reasonable diagnostic accuracy for primary tumors of ECC/ICC. According to our study, 18F-FDG PET/CT positive findings can diagnose lymph node metastases, while negative findings may not exclude the metastases. As for MRI, it can neither rule in nor rule out the disease. Therefore, 18F-FDG PET/CT may be a better choice for diagnosing lymph node metastasis of ECC/ICC. We summarized the past studies of the diagnostic value of 18F-FDG PET/CT for the staging of ECC/ICC Table 5 [12,14,16,17,29,30,31,32,33,34,35,36,37,38]. However, no past meta-analysis and few studies have evaluated the diagnostic performance of 18F-FDG PET/CT in patients with a focused-on ICC. Most studies have reported that the sensitivity, specificity, and accuracy values of 18F-FDG PET for detecting lymph node metastases were significantly higher than those of CT/MRI imaging. Compared to CT/MRI, 18F-FDG PET imaging assessments, the uptake of 18F-FDG reduces the risk of misdiagnosis, which is associated with CT/MRI and evaluations by relatively inexperienced radiologists. However, 18F-FDG PET has limitations in detecting small lesions with low tumor metabolism. The present study found that 18F-FDG PET was not superior at diagnosing satellite lesions (Figure 1) and vascular and bile duct invasion (Figure 2).

Conventional imaging techniques, such as CT and MRI, can help diagnose lymph node metastases when the lymph node size is ≥1 cm. In this study, the relationship between lymph node size and lymph node metastasis was positive. However, among patients with lymph nodes > 1 cm, 38 cases (61.3%) were positive for metastases. Among patients with lymph nodes ≤ 1 cm, 2 (7.1%) cases were diagnosed with metastasis. Thus, a lymph node size > 1 cm is not an accurate indicator of metastasis risk. Using a high SUV, 18F-FDG PET showed high sensitivity, specificity, and accuracy in detecting lymph node metastasis. Most previous studies on 18F-FDG PET for malignant tumors highlighted the modality’s capacity to detect distant metastatic lesions and tumor recurrence. These findings were validated in this study.

As a whole-body imaging technique, 18F-FDG PET is more powerful than conventional imaging techniques, such as abdominal CT/MRI. PET-CT imaging, had higher accuracy in tumor staging than CT/MRI in patients who received treatment that was different from what was originally planned. This study showed that 18F-FDG PET may help assess lymph node metastases. Moreover, this modality might be used as a complementary examination in patients with ICC alongside CT/MRI.

This study had the following major limitations. First, 105 patients, with the exception of five exploratory laparotomy cases with abdominal metastases, did not receive surgery, lacked histological staging findings, and were thus not eligible for tumor staging. In five cases, peritoneal dissemination could not be diagnosed by PET-CT, and exploratory laparotomy was performed. Therefore, the diagnostic performance of PET-CT for peritoneal dissemination seems to be limited. Second, this was not a randomized controlled study and might have been affected by selection bias. Thus, a well-designed randomized controlled trial is required to validate our findings.

## 5. Conclusions

When compared with CT or MRI, 18F-FDG PET-CT had similar sensitivity and specificity values for diagnosing satellite focus and vascular and bile duct invasion; however, PET-CT exhibited higher accuracy in diagnosing regional lymph node metastases. The accuracy of tumor staging by 18F-FDG PET-CT was higher than that by CT/MRI. Thus, 18FDG PET-CT may support tumor staging in ICC.

## Figures and Tables

**Table 1:** Clinical and histopathologic characteristics of patients undergoing surgery (n = 90).

Parameter	Case or Mean ± SD
Age (year)	64.2 (±10.6)
Sex (male/female)	57/33
Hepatitis (B/C)	8/10
CA199 (>39/≤39 U/mL)	59/31
CEA (>10/≤10 μg/L)	16/74
AFP (>20/≤20 μg/L)	6/84
Tumor location (left lobe/right lobe)	51/49
SUVmax of tumor	8.3 (3.5–14.7)
SUVmax of lymph node (>2.5/≤2.5)	32/58
Tumor size (>5/≤5 cm)	50/40
Tumor number (single/multiple)	55/35
Macroscopic vascular invasion (yes/no)	5/85
Cancer embolus in bile duct (yes/no)	6/84
Lymph node metastases (yes/no)	25/65
Nerve invasion (yes/no)	8/82
Peripheral tissue invasion (yes/no)	6/84

CA19-9—carbohydrate antigen 19-9; CEA—carcino-embryonic antigen; AFP—alpha fetoprotein; SUVmax—maximum of standardized uptake value.

**Table 2:** Diagnosis of multiple tumors, macrovascular invasion, bile duct invasion, regional lymph node metastases, and distant metastases by imaging modality.

	CT	MRI	PET-CT	CT vs. MRI vs. PET-CT	CT vs. PET-CT	MRI vs. PET-CT
Diagnosis of multiple tumors						
Sensitivity	17/35 (49%)	18/35 (51%)	10/35 (29%)	N.S.	N.S.	N.S.
Specificity	55/55 (100%)	53/55 (97%)	55/55 (100%)	N.S.	N.S.	N.S.
PPV	17/17 (100%)	18/20 (92%)	10/10 (100%)	N.S.	N.S.	N.S.
NPV	55/73 (75%)	53/70 (76%)	55/77 (71%)	N.S.	N.S.	N.S.
Accuracy	72/90 (80%)	71/90 (79%)	65/90 (72%)	N.S.	N.S.	N.S.
Diagnosis of macrovascular invasion						
Sensitivity	3/5 (60%)	3/5 (60%)	2/5 (40%)	N.S.	N.S.	N.S.
Specificity	76/85 (89%)	80/85 (94%)	83/85 (98%)	N.S.	N.S.	N.S.
PPV	3/12 (25%)	3/8 (38%)	2/4 (50%)	N.S.	N.S.	N.S.
NPV	76/78 (97%)	80/82 (98%)	83/86 (97%)	N.S.	N.S.	N.S.
Accuracy	79/90 (88%)	83/90 (92%)	85/90 (94%)	N.S.	N.S.	N.S.
Diagnosis of bile duct invasion						
Sensitivity	1/6 (17%)	3/6 (50%)	1/6 (17%)	N.S.	N.S.	N.S.
Specificity	83/84 (99%)	83/84 (99%)	84/84 (100%)	N.S.	N.S.	N.S.
PPV	1/4 (25%)	3/6 (50%)	1/1 (100%)	N.S.	N.S.	N.S.
NPV	83/84 (99%)	83/84 (99%)	84/89 (94%)	N.S.	N.S.	N.S.
Accuracy	84/90 (93%)	86/90 (96%)	85/90 (94%)	N.S.	N.S.	N.S.
Diagnosis of regional lymph node metastases						
Sensitivity	10/25 (40%)	14/25 (56%)	21/25 (84%)	0.02	<0.01	<0.01
Specificity	50/65 (80%)	54/65 (83%)	56/65 (86%)	N.S.	N.S.	N.S.
PPV	10/16 (63%)	14/19 (74%)	21/23 (91%)	0.04	0.02	N.S.
NPV	50/74 (68%)	54/71 (76%)	56/67 (84%)	<0.01	<0.01	0.02
Accuracy	60/90 (67%)	68/90 (76%)	77/90 (86%)	<0.01	<0.01	0.01

N.S. = no significant.

**Table 3:** Tumor stage assessed by CT, MRI, and PET-CT.

Stage	CT	MRI	PET-CT	Pathological Examination
I	75	70	77	56
II	5	4	2	7
III A	0	2	0	2
III B	10	14	21	25
IV	0	0	0	0

**Table 4:** Changed tumor staging assessed by CT, MRI, and PET-CT, respectively.

	Up Stage	Down Stage	Unchanged	The Accuracy
CT	10	33	47	47/90 (52%) *
MRI	9	30	51	51/90 (57%) **
PET-CT	3	19	68	68/90 (76%) ***

* *p* (CT vs. PET-CT) = 0.001, ** *p* (MRI vs. PET-CT) = 0.007, *** *p* (CT vs. MRI vs. PET-CT) = 0.003.

**Table 5:** The principal characteristics and diagnostic performances on PET-CT of past studies.

Study	Year	Country	Location	No. of Patients	Female/Male	Median Age	Study Design	Examination	Reference Standard	Primary Tumors (T)		Lymph Node Metastases (N)			Distant Metastases (M)		
										Sens.	Spec.	Sens.	Spec.	Acc.	Sens.	Spec.	Acc.
present study	2022	Japan	intrahepatic	90	33/57	64	R	CI vs. PET/CT	HP	29.0%	100.0%	84.0%	86.0%	86.0%	NA	NA	NA
Li	2018	China	hilar	53	17/36	68	R	PET/CT	HP	100.0%	NA	67.9%	88.0%	77.4%	47.1%	97.2%	81.1%
Ma	2018	China	All	66	28/38	66.0	R	PET/CT	HP	NA	NA	81.8%	NA	NA	NA	NA	NA
Lee	2017	Korea	intrahepatic	76	19/57	68	R	CI vs. PET/CT	HP	NA	NA	74.5%	90.0%	76.9%	NA (higher than CI)		
Jiang	2016	China	intrahepatic	65	NA	69.2	NA	MRI vs. PET/CT	HP	NA	NA	70.0%	91.7%	81.8%	NA	NA	NA
Adachi	2015	Japan	intrahepatic	47	NA	71	R	PET/CT	HP	NA	NA	31.2%	96.1%	NA	NA	NA	NA
Choi	2013	Korea	extrahepatic	34	NA	NA	R	PET/CT	HP	88.2%	100.0%	89.7%	NA	NA	NA	NA	NA
Ruys	2011	Netherlands	hilar	30	16/14	62	R	PET/CT	HP	88.0%	NA	67.0%	68.0%	NA	33.0%	96.0%	NA
Seo	2008	Japan	intrahepatic	35	NA	NA	R	CI vs. PET/CT	HP	NA	NA	43.0%	100.0%	86.0%	NA	NA	NA
Kim	2008	Korea	All	123	43/80	60	P	CI vs. PET/CT	HP	84.0%	79.3%	32.0%	NA	75.9%	58.0%	NA	88.3%
Li	2008	Germany	hilar	17	6/11	62	R	PET/CT	HP	58.8%	NA	41.7%	80.0%	NA	55.6%	87.5%	NA
Petrowsky	2006	Switzerland	All	61	NA	NA	P	CT vs. PET/CT	HP	55.0%	NA	12.0%	NA	NA	100.0%	NA	NA
Kim	2003	Korea	intrahepatic	21	10/11	57	R	CI vs. PET/CT	HP	NA	NA	NA (higher than CI)			NA (higher than CI)		
Kato	2002	Japan	extrahepatic	30	9/21	68	NA	CT vs. PET/CT	HP	NA	NA	38.0%	100.0%	73.0%	NA	NA	NA
Kluge	2001	Germany	All	46	21/25	63	R	CI vs. PET/CT	HP	92.3%	92.9%	13.0%	NA	NA	70.0%	NA	NA

HP = histopathology, NA = not available, No. = number, P = prospective, R = retrospective, All = intrahepatic, hilar, gallbladder and common bile duct, CI = conventional imaging, Sens. = sensitivity, Spec. = specificity, Acc. = accuracy.

## Data Availability

The data presented in this study are available on request from the corresponding author. The data are not publicly available owing to privacy.

## Supplementary Materials

The following supporting information can be downloaded at: https://www.mdpi.com/article/10.3390/diagnostics12112889/s1, Figure S1: Flow chart of patient selection.
