# Supplementary figures and images for: Comparison of Conventional Imaging and 18F-Fluorodeoxyglucose Positron Emission Tomography/Computed Tomography in the Diagnostic Accuracy of Staging in Patients with Intrahepatic Cholangiocarcinoma
Source: Diagnostics (Basel). 2022 Nov 21;12(11):2889. doi: 10.3390/diagnostics12112889 (PMC9689116; doi:10.3390/diagnostics12112889)

## Supplementary Figure S1. Flow chart of patient selection.

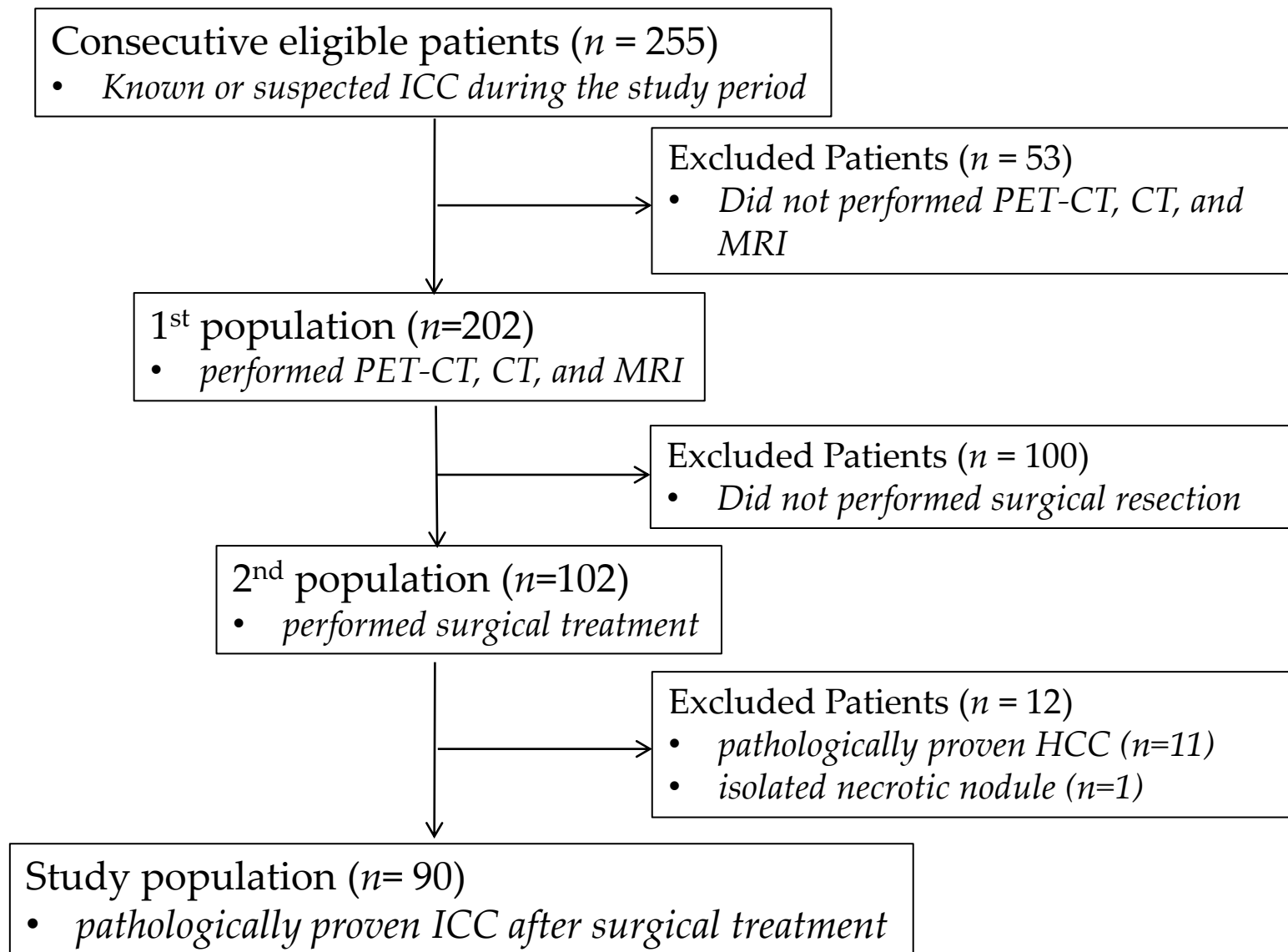

Supplement: Supplementary file 1 [file diagnostics-12-02889-s001.zip › diagnostics-1971695-supplementary.pdf]
